# The dental demolition derby: bruxism and its impact - part 2: early management of bruxism

**DOI:** 10.1038/s41415-022-4249-z

**Published:** 2022-05-27

**Authors:** Mark L. T. Thayer, Rahat Ali

**Affiliations:** 41415113427001 Consultant and Honorary Lecturer in Oral Surgery, Liverpool University Dental Hospital, Pembroke Place, Liverpool, L3 5PS, UK; 41415113427002 Consultant in Restorative Dentistry, Liverpool University Dental Hospital, Pembroke Place, Liverpool, L3 5PS, UK

## Abstract

Bruxism is a term that encompasses a range of presentations of rhythmic and repetitive muscular activity. For many, this is not a significant problem but for some, the behaviour leads to significant problems and extensive tissue damage. This is different to temporomandibular disorders. This paper will review methods of managing cases where bruxism is destructive, or potentially destructive, before needing to resort to full reconstruction.

## Key points

- Bruxism is challenging to control. The aim for early management is to limit tissue damage at an early stage rather than await extensive destruction that is then difficult to manage.
- There is also a significant financial burden to delaying intervention.
- Interventions at this stage range from pharmacological to splint therapy. Guidance on splint choice and design is provided.

## Introduction

In the first paper, the authors have reviewed the nature of bruxism and the impact this can cause. For destructive bruxism, it is difficult to identify a specific cut-off point for intervention; however, practitioners will see patients over time and with appropriate assessment, undertake suitable interventions. Management of what is a dynamic problem must also, therefore, be dynamic. To aid the decision for interventions, an intervention stratification matrix document has been introduced and this will be referred to. It is important to acknowledge that this matrix is aimed to be a support to clinical decision-making, not a driver to prescribe, nor a guideline for slavish adherence. The authors would like readers to see this as a wake-up call for those at risk.

## Management rationale

If the problem of destructive bruxism is considered as a dynamic process, then the management will also be responsive to these changes. It is important to recognise that this will present with tooth wear in various forms - or stages of development. Because bruxism is more than 'just tooth grinding', management is a long term process and there is no quick fix. Patient factors, as described in the first paper, may vary and consequently, risk assessment may vary, depending upon the point in the cycle that the patient presents. It is also important to appreciate that early assessment does not indicate long-term changes or outcomes but does provide an opportunity for baseline measurements and preventive advice and if necessary, simple interventions.

Practitioners should also be aware of the concept of 'shared care'. Here, the patient's management is divided between the general dental practitioner and the specialist. Diagnosis and treatment planning may be undertaken by one (specialist) practitioner, but the treatment might be undertaken by the patient's general dental practitioner who would also monitor the patient over time.

A 'wait and see' approach, delaying management of destructive bruxism, cannot be recommended. The concept driving management of this problem should be to never allow the patient to develop severe tooth wear due to their persistent bruxing and the authors contend that it is far superior to intervene early and limit damage to the tissues with simple interventions such as removable occlusal coverage, rather than await developments to the point where severe tooth tissue loss has occurred with its attendant difficulties and costs in reconstruction. Unfortunately, for some, presentation for management comes too late.

As a consequence, management of bruxing to protect the tissues from damage will be discussed as an integral part in the long-term management overview of these cases and this aspect will be considered in this paper.

## Routine examination

When presented with a patient who has a potential risk of tooth wear from bruxism, the core risk assessment aims to direct the general direction of management. Initial identification of key issues, such as persistent bruxing (awake or sleep) and risk environments, such as an acid-rich diet, can be made at routine examination. If a potential risk is identified, then the intervention stratification matrix can indicate the likely risk level for continued or future damage to the tissues and need for intervention. It should be noted, however, that there is a significant difference between those at risk of tissue damage and those who show simple physiological wear consistent with age, which calls for a little common sense in application of the intervention matrix (Table 1).

**Table 1:** Tooth wear and bruxism intervention need stratification

Risk factor	Score 1	Score 2	Score 3	Outcome
Age	>40	-	<40	-
Bruxing history	N	-	Long standing Childhood	-
Present bruxing	Infrequent	-	Common	-
Disturbed sleep patterns	N	-	Y	-
Extent of attrition/tooth surface loss	Low	Medium	Extensive	-
Fractures of teeth/restorations	N	-	Y	-
Number of fractures of posterior teeth/restorations (only score if above = Y)	<3	-	>3	-
Soft tissue changes/injury	N	-	Y	-
GORD	N	-	Y	-
Psychological status	Low impact	-	High impact	-
Selective serotonin reuptake inhibitor/serotonin and norepinephrine reuptake inhibitor use	N	-	Y	-
Dietary influences	N	-	Acid rich diet	-
Smoking	N	-	Y	-
**Total**				
13-17	Low need	-	-	-
18-21	Medium need	-	-	-
>22	High need	-	-	-

## Application of the intervention need matrix

**Fig 1:**
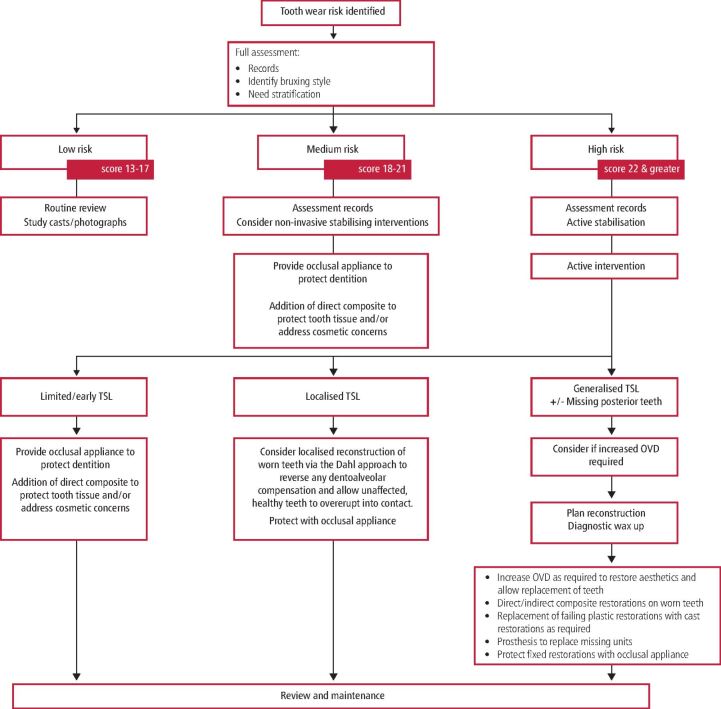
Intervention flow diagram

The matrix is completed and scored as described in paper one. This will lead to a low, medium, or high score. These scores can then guide the practitioner towards a broad treatment theme, as suggested by the application flow diagram (Fig. 1). The flow diagram summarises the rational treatment options available reflecting the level of risk and likely need for intervention.

### Low potential need

If the overall need for intervention (effectively the risk of damage) is assessed as low, then no intervention is required, but baseline records may be taken, such as diet analysis, periodontal charting, extent of any tooth wear that is present, radiographs as appropriate, study models, photographs, or a basic muscle assessment. There are a number of indices available to assess and record the degree of tooth wear a patient may present with. These include the Smith and Knight Tooth Wear Index^1^ and the Basic Erosive Wear Examination.^2^ There are also other indices that have been proposed over the years, although there is no universally accepted and adopted assessment system for a worn dentition. This may be due to a number of reasons, including the subjective nature of the assessment, failure to identify the primary cause of the tooth wear and the inability to identify small changes in hard tissue loss (that is, limited sensitivity). A review of the patient's bruxism and their tissue response can follow on a regular basis as part of the overall regular (primary care) management, without specific assessment points, unless overall assessment indicates a change to a high need for potential intervention.

### Medium potential need

Should the intervention need stratification suggest a medium risk, intervention of some form may be considered appropriate, but this is likely to take the form of stabilisation of the environment, with the primary aim being to avoid further tissue damage, as stated above. Such approaches might include (among others): fluoride applications, dietary modification, management of medical conditions such as gastro-oesophageal reflux disease (GORD) and provision of an occlusal splint that would protect the teeth from further wear. Occlusal splints should be used with caution in a potentially acid environment but may be used as a fluoride reservoir. Intervention is not, however, mandatory and should be considered in discussion with the patient. Patient engagement and education form an essential component of this part of overall management.

Attempts at comprehensive reconstruction are generally unnecessary, although some individual problems, such as a fractured cusp, may need addressing. The level of (hard) tissue loss can be limited and often inadequate to allow placement of composite (direct or indirect) or other restorations, which then suffer a high failure rate. In a number of cases, small areas of exposed dentine may, and perhaps should, be sealed with composite to manage sensitivity, especially in the context of an erosive oral environment.

### High potential need

When assessment suggests a high risk, then additional aspects of assessment should be included, with careful assessment of muscular activity, muscle tenderness and hypertrophy, pain mapping and occlusal wear patterns. Assessment of the patient's mental health may also be an important indicator and an assessment such as the Hospital Anxiety and Depression Scale is a quick, simple and validated method for this.^3^ Quality of life assessment can also guide decision-making when determining interventions. Tooth wear can have a negative impact on the patient's quality of life, which is comparable to that of being completely edentulous.^4^ The dental rehabilitation of a patient who bruxes can be difficult; the excessive occlusal forces can prematurely damage restorations and appliances, which will need regular repair and maintenance. The materials that may be better suited to such 'hostile' occlusal circumstances will be discussed later. Following assessment, initial stabilisation may be appropriate if specific factors can be identified, for example, GORD.

Formal reassessment of the dentition, with the support of the intervention need stratification matrix, may then suggest a more interventionist approach. The real challenge is determining the point of intervention. It is reasonable to argue that once the need for intervention is clear, this should not be unnecessarily delayed, as extensive destruction of the dentition is more difficult to reconstruct and much more expensive to the patient or health service, with a higher rate of failure and progressively more extensive restorations. Indeed, there is a good argument that to save the NHS extensive investment in reconstructing damaged dentitions, it would be logical to intervene early with protective occlusal appliances in order to reduce future costs. Provision of a hard acrylic occlusal appliance cost is in the region of £250-£500+, including the cost of clinical and manufacturing time, with limited ongoing costs (dependent on the level of monitoring). The cost for extensive restoration of a damaged dentition is very difficult to quantify but is significant and recurrent. Patients may need to be rehabilitated with composite resin, onlays, crowns, implant fixtures and removable overlays, onlays or overdentures. The number of teeth being treated, the material being used to restore the tooth and the clinician and laboratory technician's overhead costs will all impact on the final financial cost to the patient, which can extend to thousands of pounds. There will inevitably be recurring ongoing costs following reconstruction, with restoration fracture or failure, de-vitalisations, tooth loss and further reconstructions all being possible sequelae in such a hostile occlusal environment. The clinician must be mindful of this when treatment planning for a patient with a severely damaged dentition, secondary to bruxism.

## Interventions

Interventions take two broad approaches: preventive and reparative (reconstruction). However, whichever approach is pursued, the concept of '*primum non nocere'* and protection of the patient and the dental tissues must be observed.

### Preventive interventions

**Fig. 2:**
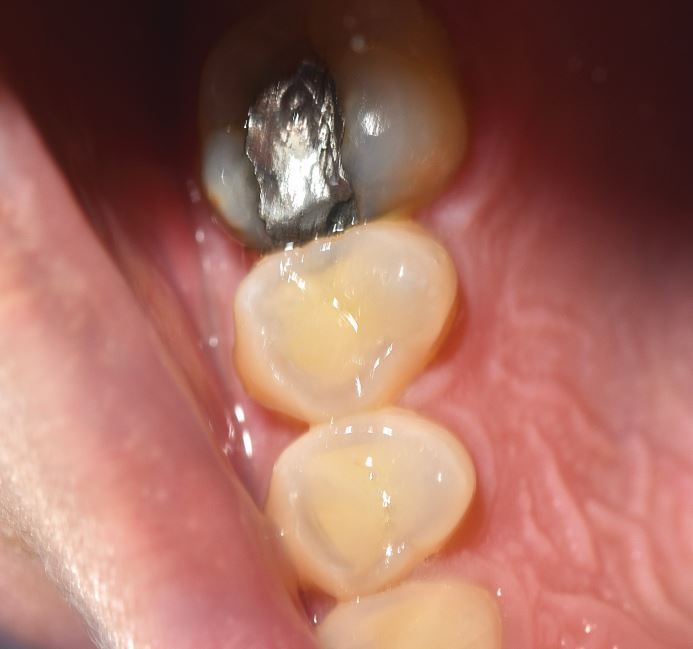
Selective (specific grinding style) wear of 14 and 15 buccal cusps in 25-year-old bruxist with intervention score of 25. Note superficial appearance of erosion is not confirmed when compared to 16 which shows no damage

Preventive interventions seek to modify and reduce muscle activity and/or provide protection of the tissues. These would be instigated when there is early but clear risk of tissue damage, ie a score of greater than 22 on the intervention matrix (Figure 2 [this patient scores 25 on intervention matrix]) but should also be considered with a medium risk score (score 18-21).

#### Reduction of muscle activity

The increased frequency and force of muscle activity is the vector for (destructive) bruxism and thus tooth wear. Control of this will reduce or stabilise symptoms and limit damage. As previously discussed, this is centrally driven, therefore options to control this can be limited.

#### Pharmacological

Given the increasingly clear link between antidepressant medication and bruxism, modification of a medication regime may be appropriate, especially if a clear association can be demonstrated in the patient history. This may require dose reduction or a change in the agent used. This must, however, be approached with significant caution and in liaison with medical and psychological support. This may produce complete resolution of bruxism or may reduce the intensity and/or frequency. In some cases, addition of a muscle relaxant can be useful and there are a number of case reports on the use of buspirone^5^^,^^6^^,^^7^^,^^8^^,^^9^^,^^10^ used to control the unintended side effect of bruxism. Buspirone is a 5-HT1A receptor partial agonist and probably acts centrally on 5-hydroxytryptamine neurons to enhance dopamine levels and inhibit muscular activity in bruxing.^8^^,^^11^ Unfortunately, however, this is not consistent.

Other approaches trialled to reduce bruxism with medication are the use of clonidine, clonazepam, propranolol, amitriptyline, aripiprazole, gabapentin and bromocriptine.^12^^,^^13^^,^^14^^,^^15^ Here, the aim is to use medication to reduce the central and peripheral motor drive to the muscles. Individual case reports show success with some patients, but a systematic review is less clear and a Cochrane review in 2014^13^ found little evidence of significant improvements with most medications. Interestingly, amitriptyline, which is used in cases of resistant muscular pain in temporomandibular disorders (TMD), was ineffective in controlling bruxism, but this does not undermine its role in pain management. Huynh and colleagues^16^ also found that clonidine affected sleep and they felt reduced bruxism intensity, if not frequency. The primary issue, as is so often the case, is the lack of clear and well-designed trials.

Proton pump inhibitors (PPI) have also been identified as a potential route for management of bruxism - specifically sleep bruxism. When GORD is present, or likely to be, gastric acidification appears to act as an initiating factor for bruxism. Control of this could reduce or terminate the irritation that triggers bruxist activity, with studies showing a reduction in rhythmic mandibular activity following administration of a PPI.^17^^,^^18^ Careful assessment of the chronology of gastric irritation and bruxing activity may provide clues revealing the origin of bruxing.

Botulinum toxin (Botox) has also been suggested as a suitable approach.^19^^,^^20^^,^^21^ This may be considered from two points of view - reduction in bruxing activity, or reduction in bruxing power reducing tissue damage. Some opinions have been wary about the use of Botox in this situation;^22^ however, its use for treatment of myofascial pain is well accepted^23^ and Botox is approved by the National Institute for Health and Care Excellence for treatment of migraines,^24^ where increased and uncontrollable muscle activity is the primary issue. Indeed, in their review paper of 2017, Patel and colleagues^20^ show that Botox is effective and imply that it might be considered as an early therapy for bruxism and TMD as the effectiveness may be more significant. However, this needs consideration, as the limitations of Botox should be realised; if bruxism is a centrally driven issue, it is unlikely to control the muscle activity, which is exactly the findings of papers looking at the use of Botox.^21^^,^^25^ In this role, Botox will reduce the power of the musculature by paralysing some of the muscle fibres and generally produces initial wasting of the muscle. This will lead to reduction in bulk of the muscle and cosmetic changes, which can be an effective method of managing those changes that have resulted from muscle hypertrophy. But if the overall activity is unchanged, this will probably lead to hypertrophy of the unaffected muscle fibres and, effectively, relapse. Botox also has a limited lifespan as new acetyl choline receptors form over time.^26^ Consequently, patients will require retreatments and the eventual development of antibodies renders Botox ineffective. In the final analysis, Botox may be best considered a rescue treatment when symptoms - primarily of muscle pain - become too severe to control with other therapies.

### Behavioural interventions

Approaches to control bruxism that have an opportunity to modify behaviour should offer greater potential for long-term management, as patient engagement is a key aspect of overall management. In fact, diagnosis and patient education form the core of all other management approaches, as without full patient engagement, interventions are undermined, compliance is inadequate, treatments fail and patient expectations are unrealised, with a potential for conflict between patient and practitioner.

Such approaches may involve classic behavioural management approaches, such as cognitive behavioural therapy (CBT), hypnotherapy and biofeedback. However, it may be that these approaches are only effective with awake bruxism, where conscious activation of the modified behaviour patterns can occur. Biofeedback has particular benefits with awake bruxism, as it gives direct control to the patient. CBT and hypnotism aim to modify centrally driven behaviour patterns. CBT can be especially useful when depressive illness or trait anxiety are present and overall management of mental health may reduce the central drive to brux, whereas hypnotism may be more useful for patients without mental health issues where central 'reprogramming' of habitual activity is the aim. Results are mixed and have strong patient-driven outcomes; however, in a systematic review, Zhang *et al.*^27^ reported that hypnosis appeared to reduce maximal pain and increase opening in TMD, with subjectively improved patient subjective evaluation of the treatment, that is, general improved positivity to their symptoms and better coping. Evidence in the review was limited, however.

Biofeedback acts to modify behaviour by demonstrating muscle activity and engaging various counter-activities to control this. Logically, this should be most effective for awake bruxism. Electromyography generally forms the basis for this, to allow the patient to appreciate muscle activity classically via a visual or audible interface and then consciously reduce the muscle activity. Evidence of effectiveness is patchy,^28^ but a recent study by Bergmann *et al.*^29^ demonstrated effective reduction in bruxing activity and symptoms with a biofeedback splint. GrindCare is a biofeedback device that uses low voltage electrical stimulus to the temporalis muscle in order to interrupt the muscle activity and induce relaxation. In a trial by Needham and Davies,^30^ this device was successful in significantly reducing bruxing and pain in a group of known bruxists by 58%. Criado *et al.*^31^ found biofeedback training was also very effective in reducing pain in many subjects and in a small study, Sato *et al.*^32^ found that biofeedback administered during the day also impacted on sleep bruxism, as well as awake bruxism.

Such behavioural approaches may be supplemented with relaxation and mindfulness exercises. Progressive relaxation with tension and relaxation of muscle groups with progression through the body provides a focus that can divert attention from (awake) bruxing activity and stimulus but also teaches the patient greater insight into their muscle behaviour. A number of phone or tablet apps are available for self-directed relaxation, although initial professional guidance is important to direct the patient to the most important muscle groups to be targeted. There are also many mindfulness apps available that can provide support to self-management strategies, although there is little specific evidence of their overall effectiveness.

#### Protection of the tissues - oral appliances

A core component of behavioural management and protection of the tissues during and after reconstruction is the use of oral appliances, including occlusal splints. The commonly accepted aim of oral appliances is that these should reduce muscle activity; however, as previously alluded to, this is a challenge to achieve in this group. In the context of this paper, the equally, if not more important role of such appliances, is the protection of the dentition and associated tissues in the persistent bruxer.

There are a wide range of appliances and each has its vociferous proponents. In a recent review of the role of occlusal appliances in managing pain in TMD and bruxism, Riley and colleagues^33^ found that despite analysing 37 trials, there was inadequate evidence to confirm the effectiveness of oral appliances. However, there were no studies reviewing the use of appliances in the control of tooth wear. The decision to use an appliance will ultimately be made on a clinical and pragmatic basis following a risk assessment. The prescribing of appliances does have a logical form but may require imagination to achieve a satisfactory result, rather than dogmatism. Broadly, there seems little benefit of an upper over a lower design, but many patients seem to find lower appliances more tolerable.

The first point to consider when prescribing an appliance is the diagnosis: is the patient presenting with sleep bruxism, awake bruxism, or both? Conventional full arch appliances are difficult to use in daytime, although some exceptionally tolerant patients will manage.

**Fig. 3:**
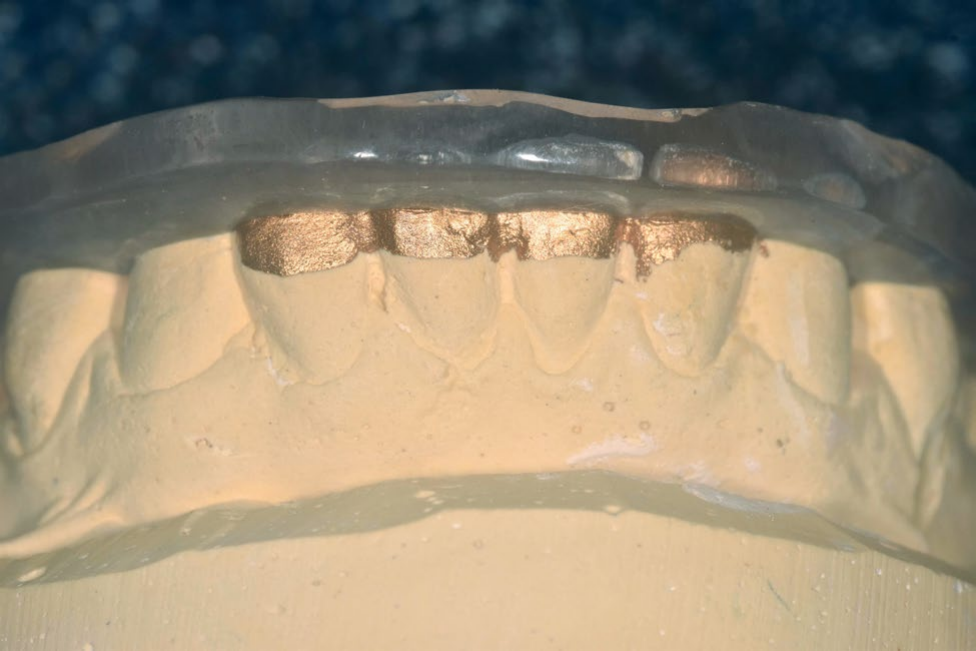
Dye paint on lower incisors of working cast
- A balanced occlusion
- Guidance conforming to the patient's natural guidance
- Include edentulous areas where appropriate (Fig. 4). Single missing teeth do not require inclusion as acrylic infill of spaces leads to increased difficulty in fitting.

Patients presenting with sleep bruxism are typically provided with an occlusal appliance to wear overnight. Adaption to appliances is usually good, but there are a number of crucial aspects to ensure adaption and compliance:

- Hard acrylic is the material of choice. Soft occlusal appliances are inappropriate as their resilience encourages muscular activity, as Oekson demonstrated in a simple but very clear crossover trial.^34^ This is reinforced by anticipatory positive feedback from soft materials (foods) encouraging higher occlusal forces than hard materials. Acceleration/deceleration speeds also influence bite force - hard materials (that is, acrylic) provide rapid deceleration force stimulus to mechanoreceptors in the periodontal ligament and masticatory muscle spindles, leading to negative feedback to the central sensory apparatus, inhibiting bite force.^35^^,^^36^^,^^37^^,^^38^ Consequently, soft appliances wear very rapidly and can be frustrating to patients as they are seemingly ineffective, although they may provide temporary protection to the tissues. Bilaminar splints may seem an ideal compromise, but the resilient fitting surface allows bruxists to crush the hard outer shell into the resilient material and failure is often rapid
- Even fit without pressure on anterior teeth and minimal active retention - usually from overall fit of acrylic to the teeth. Cribs are unnecessary. Dye paint on the incisal edges during manufacture of hard acrylic appliances helps with comfort as the incisors are very sensitive to the fit of the acrylic (Fig. 3)

**Fig. 4:**
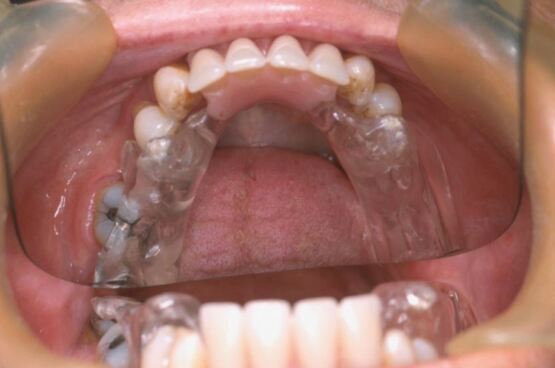
Partially dentate appliance

**Fig. 5:**
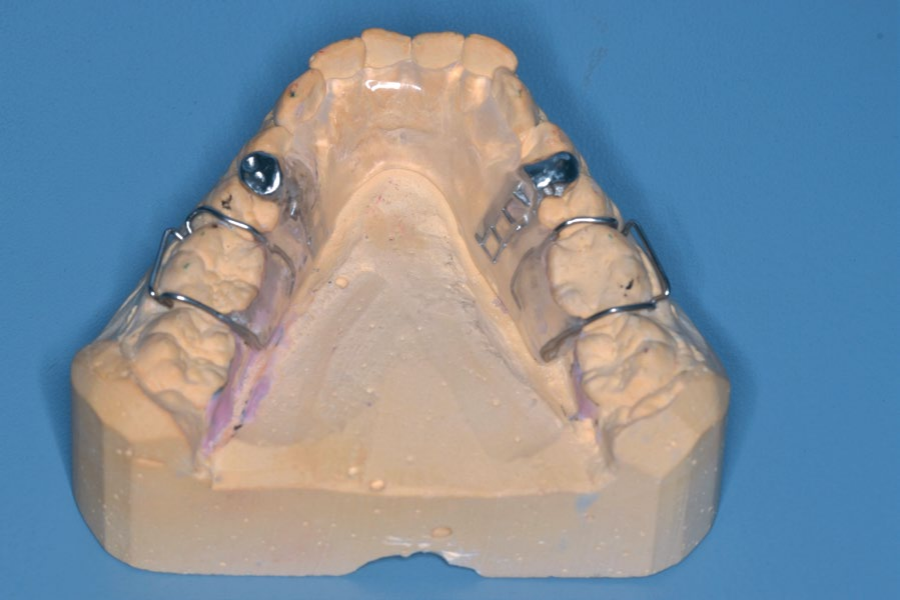
Daytime habit modifier

**Fig. 6:**
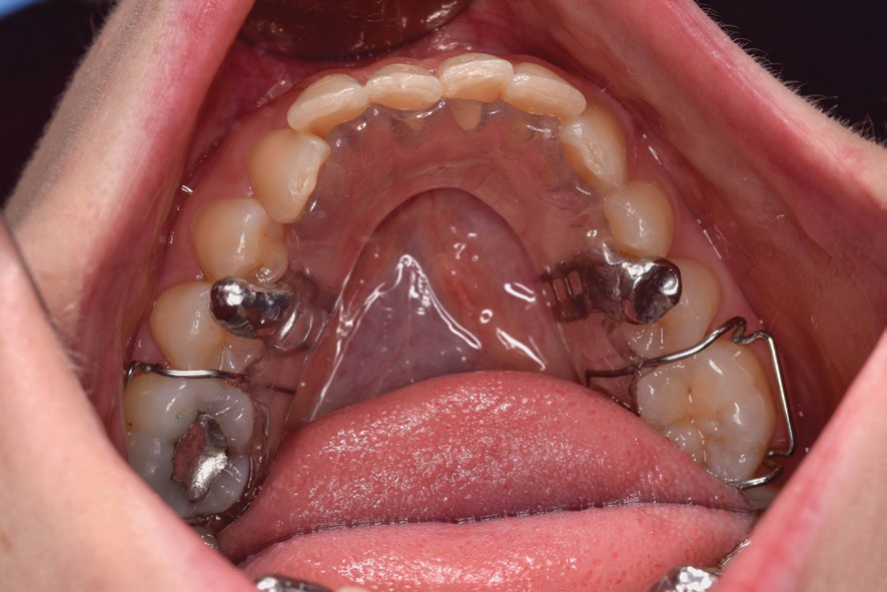
Daytime habit modifier *in situ*

For those presenting with awake bruxing, behavioural modifiers in the form of habit-breaking appliances can be extremely effective (Figures 5 and 6). Here, an interference is placed on the occlusal surface of the lower teeth to inhibit the bruxism and effectively relies upon nociceptive activation in the periodontal ligament.

Occasionally, the occlusal arrangement may be very haphazard and require intervention in both arches, with occlusal blocks replacing edentulous areas. Provision of partial dentures may also be helpful in such cases, although a review of prescribing prostheses in management of bruxing and TMD^39^ refutes the long-held view that replacement of missing posterior support will control symptoms. In some very rare circumstances, 24 hour intervention is required with both night time and daytime appliances to moderate significantly painful muscle activity.

**Fig. 7:**
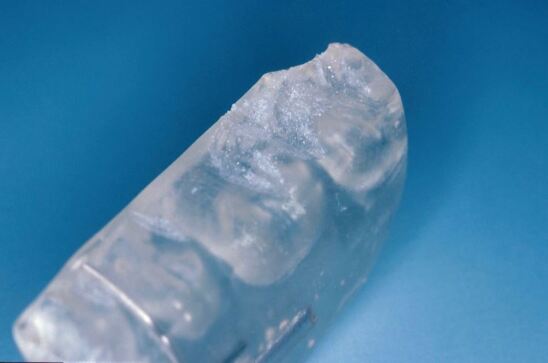
Marked wear faceting from persistent bruxism

**Fig. 8:**
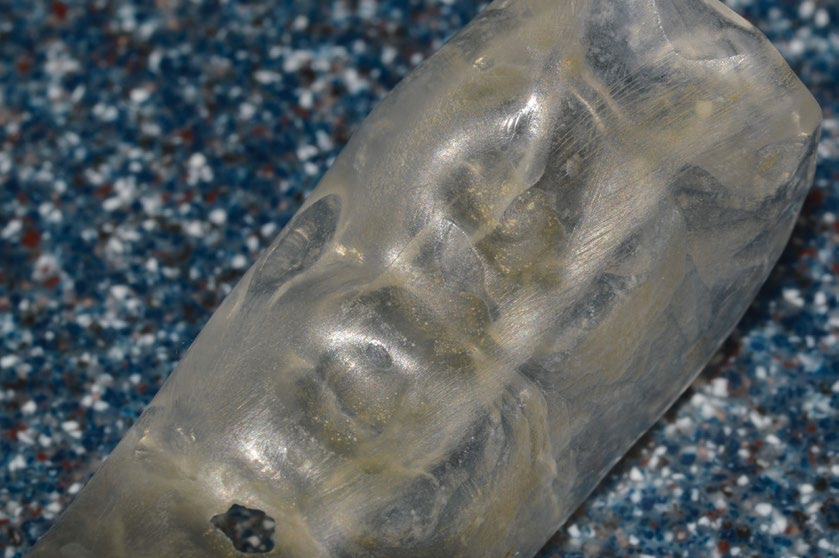
Marked wear and crazing of acrylic

**Fig. 9:**
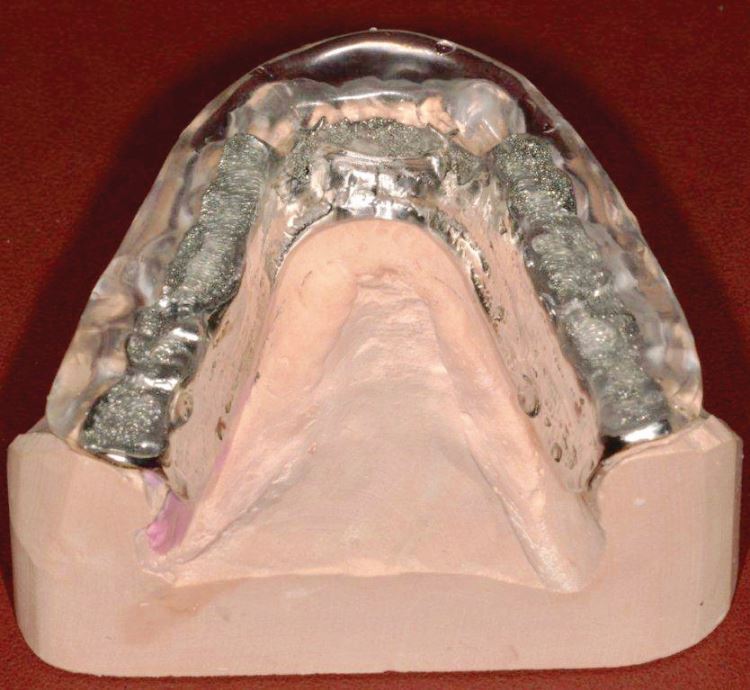
CoCr baseplate to Tanner style appliance

The second point to consider is the expected duration of treatment: is this a short-term (6-12 months) or long-term (>12 months) issue? In the context of this paper, bruxing is a chronic issue that is challenging to manage and all cases will require greater than 12 months of treatment. Forces exerted on occlusal appliances are substantial and acrylic will wear rapidly for severe bruxists and frequently crack. Figures 7 and 8 show damage to acrylic over three months of wear with active bruxing. Force applied during bruxing can exceed 900 N (or approximately double normal maximal occlusal force)^38^^,^^40^ and fracture of acrylic may occur. For those cases where forces are high and wear is rapid, a cobalt-chrome (CoCr) base framework may be required to resist the forces applied to the appliance and avoid repeated fracture of the acrylic. Wear is monitored and the acrylic can be 'resurfaced' as necessary without disturbing the fit and comfort of the appliance. The overall performance of a CoCr base seems superior to simple acrylic (Fig. 9).

**Fig. 10:**
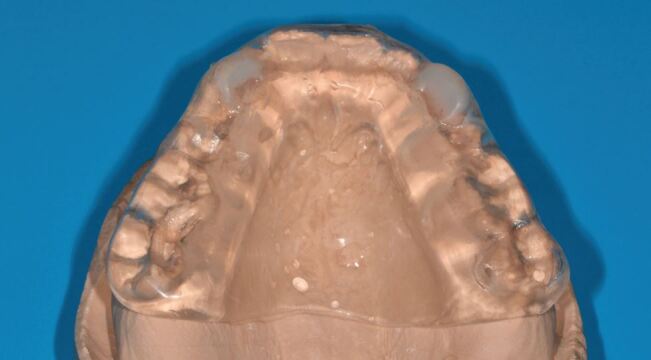
Michigan appliance risers re-enforced with composite

An alternative for controlling rapid wear of acrylic is to inset a material in the acrylic base that will show superior wear resistance - primarily composite resin (Fig. 10). A CoCr surface is undesirable as it will lead to preferential wear of opposing enamel and is immensely difficult to adjust. Microfilled composite, however, can be adjusted reasonably easily and can be replaced as a chairside repair.

The third point to consider is the occlusal arrangement: deep overbites in Class II occlusions create difficulties with appliances. Canine guidance is typically missing in Class II patients. This can lead to problems with provision of full arch coverage as the disclusion of the teeth needs to be so substantial that the appliance becomes unusable. Here, the Gelb-style appliance, with molar capping only, can be very effective. It is simple to manufacture and fit and very well tolerated. Night time wear only for management of sleep bruxism means that changes in occlusal position do not occur.

**Fig. 11:**
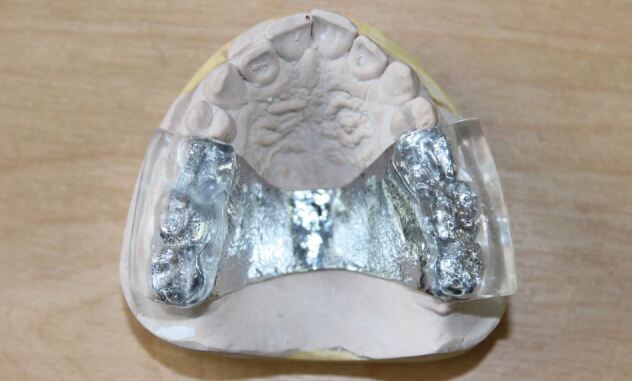
Buccal overbuild to deflect buccal tissues from the teeth

The protection of soft tissues may also be considered in the design. Soft tissues may be damaged as a result of bruxing where the buccal tissues are vulnerable to being trapped between the teeth. Simple occlusal appliances may not deflect the soft tissues away from the teeth adequately and trapping may still occur. Buccal overbuild can be included to keep the soft tissues away from the teeth (Fig. 11). Lip bumpers can be used to protect the labial mucosa.

Manufacture of the appliances is perfectly reasonable with simple occlusal records on the retruded axis at the desired vertical height of the splint and freeplane articulation. Facebow recording is unnecessarily time-consuming and the appliance should be adjusted in the mouth. In reality, the patient's bruxing will grind the occlusion in anyway! Anecdotally, the provision of a hard acrylic appliance is often seen as 'too difficult', yet the fitting is really no different to that of a denture, with simple adjustments to create a balanced occlusion.

Following fitting of the appliance, patients should be reviewed regularly: an initial review at six weeks, followed by a further review - three months is a reasonable timescale. Review is important to ensure compliance, identify issues, check effectiveness and assess performance of the appliance. Practitioners should be able to monitor progress and identify problems, such as failing restorations or excessive wear on occlusal appliances.

## Conclusion and outcomes from preventive strategies

The interventions considered in this paper aim to control and limit the destructive effects of bruxism. There is no single approach that works for all, but appliance therapy probably forms the core of this stage of management for destructive bruxism. The decision to utilise various strategies outlined relies upon effective listening to the patient behaviours and interpretation of bruxing drivers. Prescribing of management approaches might be described as 'cherry picking' to identify the mix of approaches that will improve control of bruxism. The use of occlusal appliances varies and is dependent on the interventional rationale. If the aim is to reduce bruxism, with or without muscular pain, then the ideal outcome will be symptom control. However, if the primary driving force for bruxism is central, as discussed in the first paper, then elimination of bruxism is unlikely (but not impossible). Habit breaker appliances worn during the day to control bruxism are extremely effective in most cases, as they redirect control to the patient.

In reality, the outcomes should be the protection of the tissues, hard or soft, and restorations, be they amalgam, ceramic, or implant-retained. In such cases, results will become apparent over time: a reduction in trauma to soft tissues, fewer fractures, reduced speed of tooth tissue loss, fewer fractured restorations or screw-retained prosthesis mobilisation and fewer acute presentations with problems, releasing clinical time for other cases. Appliances will (generally) wear, with loss of acrylic over time but may be remade or refaced.

Oral appliances have a substantial role in the prevention and control of damage to the tissues during bruxing. Unfortunately, many patients attend without early identification and prevention strategies being in place. As a consequence, significant damage to the tissues may have occurred. In the third paper of the series, the reconstruction of the dentition will be considered in the context of the continuing bruxist.

## References

[1] Smith B G, Knight J K. An index for measuring the wear of teeth. *Br Dent J *1984; **156:** 435-438. 10.1038/sj.bdj.4805394 6590081

[2] Bartlett D, Ganss C, Lussi A. Basic Erosive Wear Examination (BEWE): a new scoring system for scientific and clinical needs. *Clin Oral Investig* 2008; DOI: 10.1007/s00784-007-0181-5. 10.1007/s00784-007-0181-5 PMC2238785 18228057

[3] Stern A F. The hospital anxiety and depression scale. *Occup Med (Lond)* 2014; **64:** 393-394. 10.1093/occmed/kqu024 25005549

[4] Papagianni C E, van der Meulen M J, Naeije M, Lobbezoo F. Oral health-related quality of life in patients with tooth wear. *J Oral Rehabil *2013; **40:** 185-190. 10.1111/joor.12025 23278167

[5] Ranjan S, Chandra P S, Prabhu P. Antidepressant-induced bruxism: need for buspirone? *Int J Neuropsychopharmacol *2006; **9:** 485-487. 10.1017/S1461145705005985 16753073

[6] Kuloglu M, Ekinci O, Caykoylu A. Venlafaxine-associated nocturnal bruxism in a depressive patient successfully treated with buspirone. *J Psychopharmacol *2010; **24:** 627-628. 10.1177/0269881109102612 19264817

[7] Albayrak Y, Okan Ekinci O. Duloxetine-induced nocturnal bruxism resolved by buspirone: case report. *Clin Neuropharmacol *2011; **34:** 137-138. 10.1097/WNF.0b013e3182227736 21768799

[8] Sabuncuoglu O, Ekinci O, Berkem M. Fluoxetine-induced sleep bruxism in an adolescent treated with buspirone: a case report. *Spec Care Dentist* 2009; **29:** 215-217. 10.1111/j.1754-4505.2009.00091.x 19740153

[9] Bostwick J M, Jaffee M S. Buspirone as an antidote to SSRI-induced bruxism in 4 cases. *J Clin Psychiatry *1999; **60:** 857-860. 10.4088/jcp.v60n1209 10665633

[10] Milanlioglu A. Paroxetine-induced severe sleep bruxism successfully treated with buspirone. *Clinics (Sao Paulo) *2012; **67:** 191-192. 10.6061/clinics/2012(02)17 PMC3275112 22358247

[11] Çolak Sivri R C, Akça O F. Buspirone in the Treatment of Fluoxetine-Induced Sleep Bruxism. *J Child Adolesc Psychopharmacol *2016; **26:** 762-763. 10.1089/cap.2016.0075 27315110

[12] Rajan R, Sun Y-M. Reevaluating Antidepressant Selection in Patients With Bruxism and Temporomandibular Joint Disorder. *J Psychiatr Pract *2017; **23:** 173-179. 10.1097/PRA.0000000000000227 28492455

[13] Macedo C R, Macedo E C, Torloni M R, Silva A B, Prado G F. Pharmacotherapy for sleep bruxism. *Cochrane Database Syst Rev* 2014; DOI: 10.1002/14651858.CD005578.pub2. 10.1002/14651858.CD005578.pub2 PMC11033873 25338726

[14] Carra M C, Macaluso G M, Rompré P H* et al. *Clonidine has a paradoxical effect on cyclic arousal and sleep bruxism during NREM sleep. *Sleep* 2010; **33:** 1711-1716. 10.1093/sleep/33.12.1711 PMC2982742 21120152

[15] Sakai T, Kato T, Yoshizawa S* et al. *Effect of clonazepam and clonidine on primary sleep bruxism: a double-blind, crossover, placebo-controlled trial. *J Sleep Res* 2017; **26:** 73-83. 10.1111/jsr.12442 27485389

[16] Huynh N, Lavigne G J, Lanfranchi P A, Montplaisir J Y, de Champlain J. The effect of 2 sympatholytic medication - propranolol and clonidine - on sleep bruxism: experimental randomized controlled studies. *Sleep* 2006; **29:** 307-316. 10.1093/sleep/29.3.307 16553016

[17] Miyawaki S, Tanimoto Y, Araki Y, Katayama A, Fujii A, Takano-Yamamoto T. Association between nocturnal bruxism and gastroesophageal reflux. *Sleep* 2003; **26:** 888-892. 10.1093/sleep/26.7.888 14655925

[18] Ohmure H, Kanematsu-Hashimoto K, Nagayama K* et al. *Evaluation of a Proton Pump Inhibitor for Sleep Bruxism: A Randomized Clinical Trial.* J Dent Res *2016; **95:** 1479-1486. 10.1177/0022034516662245 27474257

[19] Ondo W G, Simmons J H, Shahid M H, Hashem V, Hunter C, Jankovic J. Onabotulinum toxin-A injections for sleep bruxism: A double-blind, placebo-controlled study. *Neurology* 2018; DOI: 10.1212/WNL.0000000000004951. 10.1212/WNL.0000000000004951 29343468

[20] Patel J, Cardoso J A, Mehta S. A systematic review of botulinum toxin in the management of patients with temporomandibular disorders and bruxism. *Br Dent J* 2019; **226:** 667-672. 10.1038/s41415-019-0257-z 31076698

[21] De la Torre Canales G, Câmara-Souza M B, do Amaral C F, Garcia R C M R, Manfredini D. Is there enough evidence to use botulinum toxin injections for bruxism management? A systematic literature review. *Clin Oral Investig *2017; **21:** 727-734. 10.1007/s00784-017-2092-4 28255752

[22] Kün-Darbois J-D, Libouban H, Chappard D. Botulinum toxin in masticatory muscles of the adult rat induces bone loss at the condyle and alveolar regions of the mandible associated with a bone proliferation at a muscle enthesis. *Bone* 2015; **77:** 75-82. 10.1016/j.bone.2015.03.023 25857689

[23] Farrier J N, Farrier S, Haworth S, Beech A N. Can we justify the continued use of botulinum toxin A in the management of myofascial pain? *Br J Oral Maxillofac Surg* 2020; **58:** 1133-1138. 10.1016/j.bjoms.2020.06.024 32622616

[24] National Institute for Health and Care Excellence. Botulinum toxin type A for the prevention of headaches in adults with chronic migraine: Technology appraisal guidance [TA260]. 2012. Available at https://www.nice.org.uk/guidance/ta260 (accessed April 2022).

[25] Shim Y J, Lee M K, Kato T, Park H U, Heo K, Kim S T. Effects of botulinum toxin on jaw motor events during sleep in sleep bruxism patients: a polysomnographic evaluation. *J Clin Sleep Med* 2014; **10:** 291-298. 10.5664/jcsm.3532 PMC3927435 24634627

[26] Sellin L C, Thesleff S. Pre- and post-synaptic actions of botulinum toxin at the rat neuromuscular junction. *J Physiol *1981; **317:** 487-495. 10.1113/jphysiol.1981.sp013838 PMC1246802 6273549

[27] Zhang Y, Montoya L, Ebrahim S* et al. *Hypnosis/Relaxation therapy for temporomandibular disorders: a systematic review and meta-analysis of randomized controlled trials. *J Oral Facial Pain Headache *2015; **29:** 115-125. 10.11607/ofph.1330 25905529

[28] Jokubauskas L, Baltrušaitytė A. Efficacy of biofeedback therapy on sleep bruxism: A systematic review and meta-analysis.* J Oral Rehabil *2018; **45:** 485-495. 10.1111/joor.12628 29577362

[29] Bergmann A, Edelhoff D, Schubert O, Erdelt K-J, Pho Duc J-M. Effect of treatment with a full-occlusion biofeedback splint on sleep bruxism and TMD pain: a randomized controlled clinical trial. *Clin Oral Investig* 2020; **24:** 4005-4018. 10.1007/s00784-020-03270-z PMC7544753 32430774

[30] Needham R, Davies S J. Use of the Grindcare® device in the management of nocturnal bruxism: a pilot study. *Br Dent J* 2013; DOI: 10.1038/sj.bdj.2013.653. 10.1038/sj.bdj.2013.653 23846087

[31] Criado L, de La Fuente A, Heredia M, Montero J, Albaladejo A, Criado J-M. Electromyographic biofeedback training for reducing muscle pain and tension on masseter and temporal muscles: A pilot study. *J Clin Exp Dent* 2016; DOI: 10.4317/jced.52867. 10.4317/jced.52867 PMC5149094 27957273

[32] Sato M, Lizuka T, Watanabe A* et al. *Electromyogram biofeedback training for daytime clenching and its effect on sleep bruxism. *J Oral Rehabil *2015; **42:** 83-89. 10.1111/joor.12233 25256380

[33] Riley P, Glenny A-M, Worthington H V* et al. *Oral splints for temporomandibular disorder or bruxism: a systematic review. *Br Dent J* 2020; **228:** 191-197. 10.1038/s41415-020-1250-2 PMC7718146 32060462

[34] Oekson J P. The effects of hard and soft occlusal splints on nocturnal bruxism. *J Am Dent Assoc* 1987; **114:** 788-791. 10.14219/jada.archive.1987.0165 3475357

[35] Serra C M, Manns A E. Bite force measurements with hard and soft bite surfaces. *J Oral Rehabil* 2013; **40:** 563-568. 10.1111/joor.12068 23692029

[36] Pereira L J, Duarte Gaviao M B, Van Der Bilt A. Influence of oral characteristics and food products on masticatory function. *Acta Odontol Scand* 2006; **64:** 193-201. 10.1080/00016350600703459 16829493

[37] Shimada A, Yamabe Y, Torisu T, Baad-Hansen L, Murata H, Svensson P. Measurement of dynamic bite force during mastication. *J Oral Rehabil *2012; **39:** 349-356. 10.1111/j.1365-2842.2011.02278.x 22288929

[38] Custodio W, Gomes S G F, Faot F, Garcia R C M R, Del Bel Cury A A. Occlusal force, electromyographic activity of masticatory muscles and mandibular flexure of subjects with different facial types. *J Appl Oral Sci* 2011; **19:** 343-349. 10.1590/S1678-77572011005000008 PMC4223785 21655772

[39] Manfredini D, Poggio C E. Prosthodontic planning in patients with temporomandibular disorders and/or bruxism: A systematic review. *J Prosthet Dent* 2017; **117:** 606-613. 10.1016/j.prosdent.2016.09.012 27836142

[40] Waltimo A, Nyström M, Könönen M. Bite force and dentofacial morphology in men with severe dental attrition. *Scand J Dent Res* 1994; **102:** 92-96. 10.1111/j.1600-0722.1994.tb01161.x 8016561

